# Revisits and frailty in older patients in the emergency department - a prospective observational multicenter study

**DOI:** 10.1186/s12873-024-01123-6

**Published:** 2024-10-29

**Authors:** Helena Johansson, Sara Fahlander, Erika Hörlin, Joakim Henricson, Samia Munir Ehrlington, Jens Wretborn, Daniel Wilhelms

**Affiliations:** 1 Department of Emergency Medicine, Department of Biomedical and Clinical Sciences, Linköping University, Linköping, Sweden; 2 Falck Emergency Östergötland, Linköping, Sweden

**Keywords:** Frailty, Emergency Department, Revisits, Return visits, Admission, Mortality, Clinical Frailty Scale

## Abstract

**Background:**

An increased number of revisits may signal that the immediate medical needs of patients seeking care at Emergency Departments (EDs) are not being met. The prevalence and characteristics of revisits to the EDs in Sweden among older patients, and its association to frailty, are unknown. We aimed to investigate the prevalence of ED revisits among patients over 65 years of age, living with or without frailty, and its association with rate of admission, and mortality; in the Swedish ED setting.

**Methods:**

This was a prospective, multicentre study of patients over 65 years of age with an index visit to one of three Swedish EDs during May-Nov 2021. Frailty was assessed in conjunction with standard triage, using the 9-level Clinical Frailty Scale (CFS) with a CFS score of 5 to 8 as cut-off for identifying frailty. For all patients who made a revisit within 90 days of their index visit, we collected information about the revisit, admission, and mortality.

**Results:**

A total of 1835 patients made an index visit which were included, and out of those, 595 patients made a revisit within 90 days of the index visit. Patients living with frailty (CFS 5 to 8) were more likely to make a revisit to the ED at 8 to 30 days (17% vs. 11%, diff 6%, 95% CI 2–10%, *p* < 0.001) and at 31 to 90 days (19% vs. 12%, diff 7%, 95% CI 3–10%, *p* < 0.001) and be admitted to in-hospital care during their revisit (57% vs. 47%, diff 10%, 95% CI, 1–18%, *p* < 0.05), compared to patients living without frailty. Results also show that patients living with frailty had a higher overall mortality rate (17% vs. 5%, diff 12%, 95% CI 7–18%, *p* < 0.001). However, among patients living without frailty, making a revisit slightly increased the mortality rate compared to those who did not (5% vs. 2%, diff 3%, 95% CI 1–10%, *p* < 0.05).

**Conclusions:**

Patients living with frailty make more revisits, are more often admitted to in-hospital care, and have a higher overall mortality rate than patients not living with frailty. Frailty, assessed with the CFS may be a simple and useful indicator of increased risk of adverse events, including revisits, in the ED.

## Introduction

The number of individuals aged over 65 and over visiting emergency departments (EDs) worldwide is steadily increasing, reflecting a global trend of an increasing number of older adults seeking emergency care [1]. In 2022, about 16% of Sweden’s 10.5 million population was over 65. Among all adult patients (aged 18 and above) visiting Swedish EDs, those over 65 accounted for approximately 45%.^2^ This may present a challenge to ED staff as older people often have multiple comorbidities and complex medical and care needs [2–6]. There are several known adverse outcomes for older patients seeking care at an ED, for example increased length of stay at the ED, or readmissions within 30 days [7]. As populations age and healthcare needs become more complex, requiring more in-hospital care, the number of hospital beds in all OECD countries has steadily decreased each year. In 2023, Sweden had only 2 hospital beds per 1.000 residents [8]. Regarding community care in Sweden, 290 municipalities are responsible for home care, emergency alarm, care homes and nursing homes - services utilized by almost 340 000 people ≥ 65 years of age [9]. An increased number of revisits to EDs may signal that all the medical needs of ED patients are not being met. Previous studies have, depending on the cut-off length of the investigated timeperiod from index visit to revisit, shown a range for the fraction of older patients returning to the ED between 10–49%.^7^ In Sweden, one study found that about 20% of ED visits by patients over 65 years of age resulted in a revisit within 30 days. Factors associated with these revisits included male sex, polypharmacy, being in the last year of life, and ED care utilization [10]. However, frailty was not assessed in this study.

Frailty is a condition described as a state of increased vulnerability to stressors, due to a decline in several inter-related physiological systems. This increases the risk of adverse events, such as falls, delirium and pharmaceutical side effects [11, 12]. Frailty has also been associated with higher probability for admittance to in-hospital care [13]. A visit to the ED is a stressor in itself, posing a risk to increase frailty in older patients [7, 11, 12], which potentially could increase the risk of revisits. Therefore, it is important to identify frailty during an ED visit [6, 11, 13, 14]. There is a variety of different tools used to identify frailty in the ED [6, 15], and the Clinical Frailty Scale (CFS) has been shown to be both an accurate tool in assessing frailty, as well as a workable tool in the ED [6, 15–18].

CFS is an assessment tool based on an individual’s daily functioning and cognitive status, scored on a 9-point scale ranging from 1 to 9 [14, 19]. It is one of the most evaluated frailty screening tools for use on people over 65 years of age, and is currently being used in the acute care setting all over the world [17, 18]. It has the ability to contribute to proper risk stratification of older ED patients [18, 20, 21], is fast and easy to administrate and understand, non-reliant to equipment or extensive documentation [15], and it has been shown to be a workable tool in the ED setting [21, 22]. CFS has been shown to be able to predict patient outcome after an ED visit regarding hospital length of stay, overall mortality and readmission [20, 21, 23]. Hence, it could be useful in order to individualize patient care.

Identifying patients at risk of revisits, using simple tools like frailty through the CFS, is essential to optimize and reduce the already heavy burden on the emergency care system. The prevalence and characteristics of revisits to the ED in Sweden in older patients, and its potential association to frailty is currently unknown. Therefore, the aim of this study was to investigate the prevalence of ED revisits within 90 days of index visit; the rate of admission at revisit; the mortality, in a Swedish ED setting among patients over 65 years of age, living with or without frailty.

## Methods

### Definition of revisits

The authors of this study, after revieving the literature, defined a revisit to the ED as a unique unscheduled return visit to the ED following discharge, regardless of destination, within a specific time period.

### Study design and setting

This was a prospective observational study, carried out in three EDs in south-east Sweden, with approximately 125 000 annual visits combined. One ED is in an urban tertiary care center, another is an urban community hospital, and a third is in a rural community hospital (Table 1). The three EDs serve a joint population of approximately 465 000 inhabitants. About 35% of the patient population visiting one of these three EDs is 65 years of age or older, and the overall admission rate in these EDs is about 20%. Patients ≥ 65 years of age accounts for approximately 65% of all admissions to in-hospital care. Data were collected over a six-week period in each ED (Table 1), though the time points for data collection differed between EDs due to organizational factors. Data collection was performed at all hours of the day during the study period. A staff member on the emergency care team (a physician, registered nurse, or assistant nurse) performed the frailty assessment during the patient’s stay at the index ED visit. Frailty was measured using the Swedish version of CFS [14], patients living with frailty were defined as those with a CFS score of 5 to 8. As previous studies suggest, patients assessed as CFS 9 were excluded, since they are considered terminally ill, but not necessarily frail in other aspects [14, 21].

**Table 1:** Characteristics and recruitment periods for the three participating emergency departments (EDs)

	Linköping Emergency Department	Norrköping Emergency Department	Motala Emergency Department
Annual ED visits	50 000	50 000	25 000
Type	University hospital	Urban Community hospital	Rural Community hospital
Recruiting period	6 weeks in May/June 2021	6 weeks in October/November 2021	6 weeks in October/November 2021

Data collected in this study was a part of a large research program aimed to study different aspects of the CFS in a Swedish ED-context [22]. The three EDs had recently introduced CFS to the clinical routine and most of the ED staff (77%) had completed training on the subject. The study was approved by the Swedish Ethical Review Authority (permit no. 2021 − 00875) and the need for informed consent was waived.

Participants included in the study were patients ≥ 65 years of age making one index visit at any of the three EDs within their respective recruiting period (Table 1) with a CFS-score noted on a specific worksheet. Participants were excluded if the worksheet was incomplete, if they were assessed as CFS = 9 or if the electronic health record for the index visit or return visit was missing. In the event of multiple revisits within 90 days from the index visit, only the first revisit was assessed. Data on index visits, revisits, admissions and mortality were obtained via the electronic medical records.

Recorded revisits were divided into three time periods in the analysis, 0 to 7 days, 8 to 30 days and 31 to 90 from index visit.

### Outcomes

The primary outcome was: the prevalence of ED revisits at 7, 30 and 90 days, described as the proportion (%) of revisits in relation to the number of index visits. Secondary outcomes were: the rate of admissions for patients that made a revisit within 7, 30 and 90 days, expressed as the proportion (%) of total number of admitted patients; and difference in mortality rate among patients who revisited the ED within 90 days in relation to the non-revisiting patients. Each outcome was presented for patients living with or without frailty respectively.

### Data analysis and statistics

All statistics are reported as frequencies, with mean and standard deviation (SD), or as number and percentages (%). Categorical variables were analyzed with Chi-Square-test, using Yates’s correction for continuity where applicable [24, 25]. Significance level was set at *p*-value < 0.05, and confidence intervals (CI) were calculated. Statistical analysis was done in IBM SPSS Statistics, version 29.0.1.1, and CI and *p*-value were calculated in “R”.

## Results

**Fig. 1:**
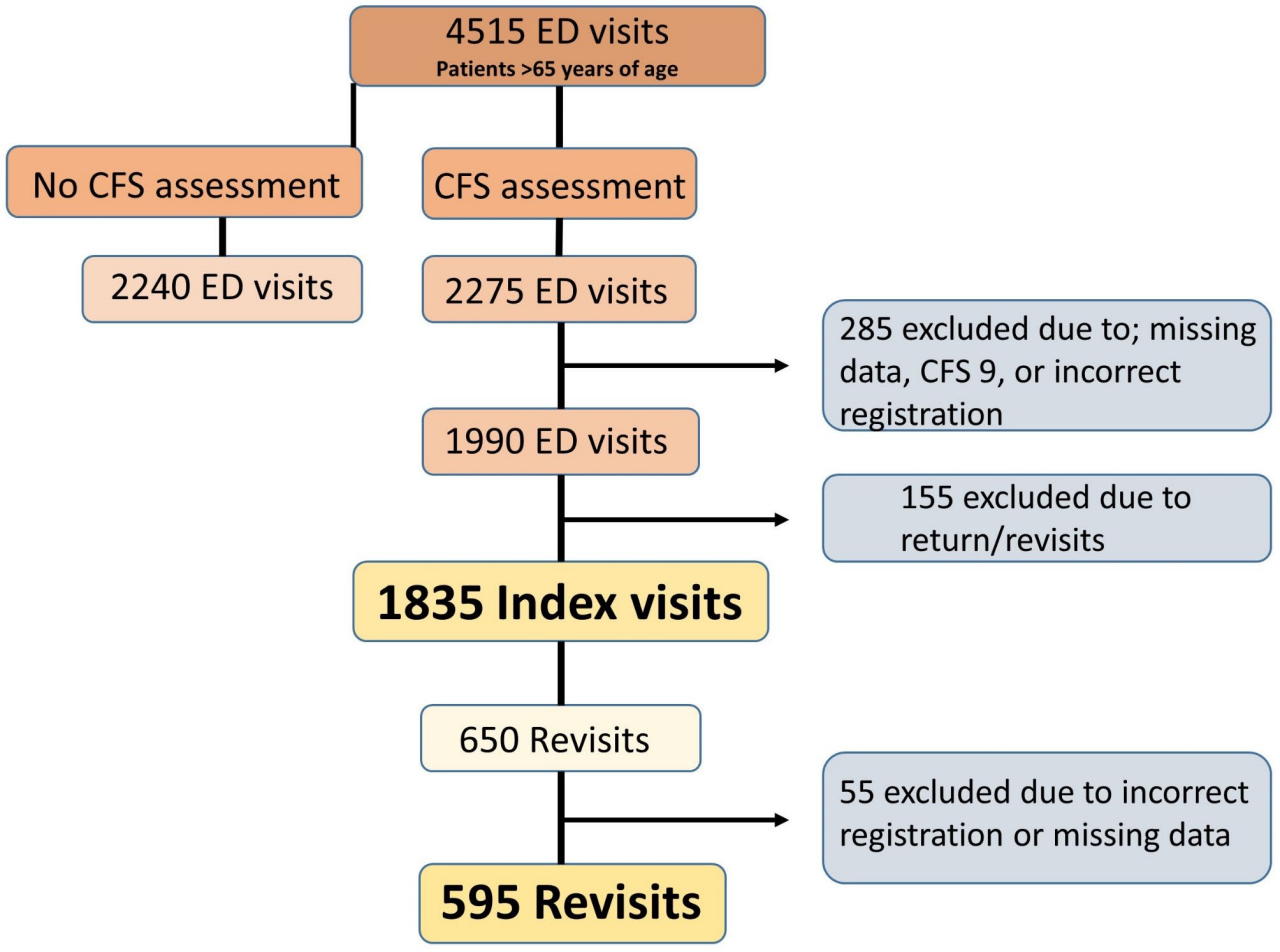
Exclusion process

In total, 4515 ED visits were made by patients aged 65 or above to any of the study sites during the data collection period. Of these, 2275 patients were assessed for frailty using CFS, and thus eligible for inclusion. A total of 440 patients were excluded since they did not meet all the inclusion criteria of the study. This led to 1835 patients making an index visit, which were included for analysis. Out of those, 650 patients made a unique revisit to the ED within 90 days. However, 55 patients were excluded due to incorrect registration or missing data in the electronic medical records, rendering 595 (32%) revisiting patients included in the study (Fig. 1).

Patients living with frailty were in minority among both revisiting and non-revisiting patients. They were also older, had a higher proportion of women and higher mortality, than patients not living with frailty (Table 2).

**Table 2:** Demographic of index cohort, divided in patients living with/without frailty and revisiting/non-revisiting patients

Revisiting patients	CFS 5–8 (living with frailty	CFS 1–4 (not living with frailty)
Total *N* (%)	244 (41)	351 (59)
Age Mean (SD)	83 (8)	78 (7)
Female *N* (%)	150 (62)	179 (51)
Deceased within 90 days *N* (%)	42 (17)	16 (5)
Deceased 0–7 days *N* (%)	1 (0)	1 (0)
Deceased 8–30 days *N* (%)	13 (5)	1 (0)
Deceased 31–90 days *N* (%)	28 (12)	14 (4)
**Non-revisiting patients**	**CFS 5–8 (living with frailty**	**CFS 1–4 (not living with frailty)**
Total *N* (%)	357 (29)	883 (71)
Age Mean (SD)	83 (8)	76 (7)
Female *N* (%)	216 (61)	458 (52)
Deceased within 90 days *N* (%)	50 (14)	13 (2)
Deceased 0–7 days *N* (%)	14 (4)	1 (0)
Deceased 8–30 days *N* (%)	18 (5)	8 (1)
Deceased 31–90 days *N* (%)	18 (5)	4 (1)

### The prevalence of ED revisits

**Fig. 2:**
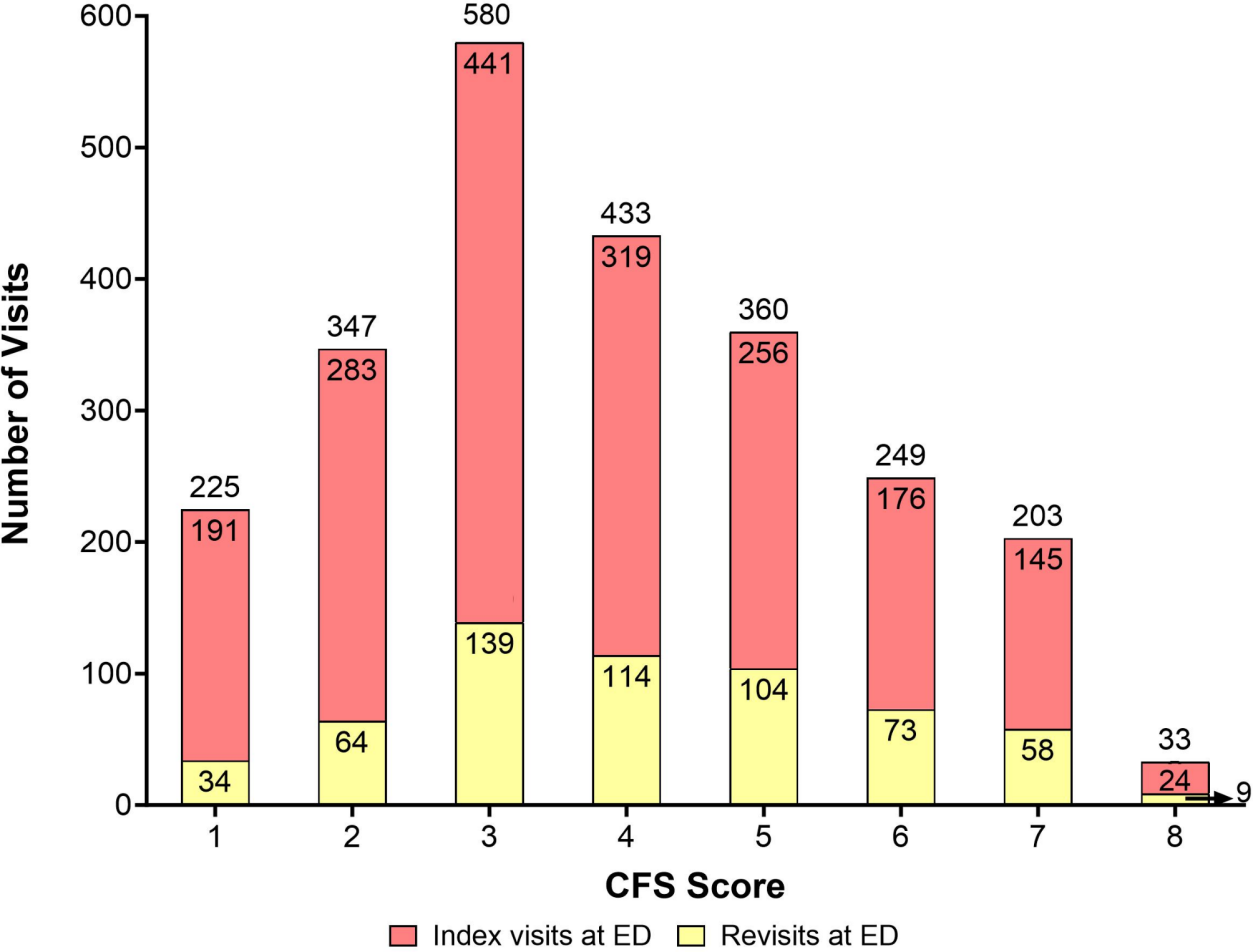
The figure shows the total number of index visits, number of non-revisiting patients, number of revisiting patients in each CFS-category respectively. CFS 8 the number of revisits (*n* = 9) is written next to the plot

Patients living with frailty had a higher proportion of revisits with approximately 40% of the patients making a revisit within 90 days, compared to patients not living with frailty (Fig. 2).

**Fig. 3:**
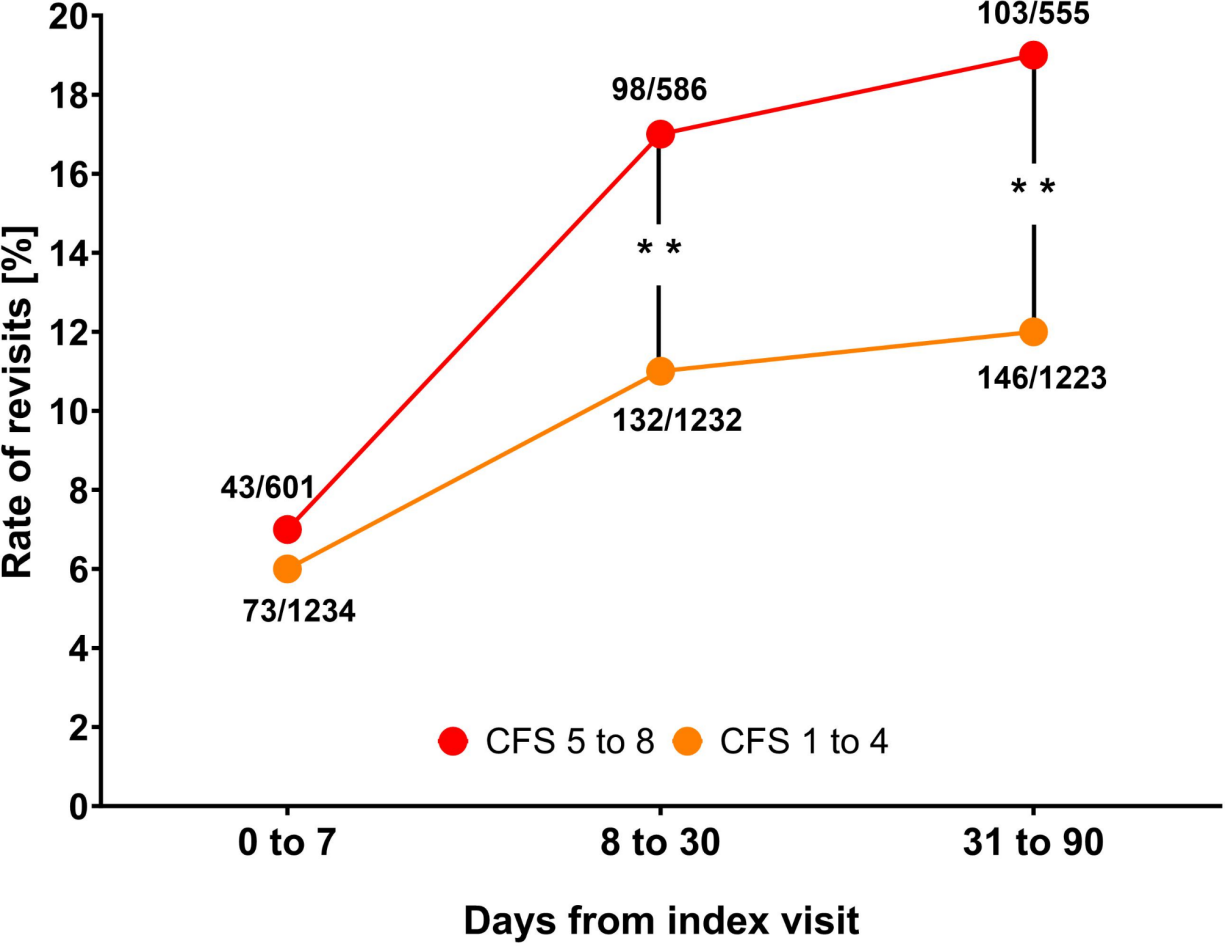
Rate of revisits divided into patients living with or without frailty, showing when in time revisits were made from index visit. Patients who were deceased in the preceding time periods were removed from the total number of patients in the frail and non-frail groups respectively

Within the first 7 days of the index visit, there was no variation in rate of revisits between the two groups (7% vs. 6%, difference 1%, *p* = 0.33). Within 8 to 30 days of the index visit, patients living with frailty made significantly more revisits in comparison to patients living without frailty (17% vs. 11%, difference 6%, 95% CI 2 to 10%, *p* < 0.001), and a similar pattern was seen for the period 31 to 90 days within index visit (19% vs. 12%, difference 7%, 95% CI 3 to 10%, *p* < 0.001), as seen in Fig. 3.

### Rate of admission at revisit

Of the 595 revisits occurring within 90 days from index visit, 305 (51%) resulted in admission. Patients living with frailty experienced a significantly higher overall admission rate during revisit, with 139 out of 244 (57%) being admitted, compared to 166 out of 351 (47%) patients living without frailty (Table 3). This trend persisted across subgroups analyzed at 0–7, 8–30, and 31–90 days following the index visit. However, it is important to note that these differences did not reach statistical significance.

**Table 3:** Admission rate at revisit for patients living with/without frailty within the respective timeframes. *=*p* < 0,05

Days from index visit	Admission Rate (%)		Diff (%)	95% CI	*p*-value
	CFS 5 to 8	CFS 1 to 4			
0 to 90	57	47	10	1 to 18	0.02*
0 to 7	58	44	14	-6 to 35	0.2
8 to 30	53	47	6	-8 to 20	0.4
31 to 90	60	49	11	-2 to 24	0.1

### Mortality

Patients living with frailty presented significantly higher 90-day mortality rates compared to those living without frailty, regardless of whether they revisited the ED or not.

In the non-revisit group (*n* = 1240), 50 out of 357 (14.0%) patients living with frailty died, compared to 13 out of 883 (1.5%) patients living without frailty (difference 12.5%, 95%CI 7 to 18, *p* < 0.001). In the revisit group (*n *= 595), 42 out of 244 (17.2%) patients living with frailty died, compared to 16 out of 351 (4.6%) patients living without frailty (difference 12.6%, 95%CI 9 to 16, *p* < 0.001).

For patients living with frailty, revisits did not significantly impact mortality rates (17% for revisits vs. 14% for non-revisits, difference: 3%, *p* = 0.38). However, among patients living without frailty, those who revisited the ED had a slightly higher mortality rate compared to those who did not (5% vs. 2%, difference: 3%, 95% CI: 1 to 6, *p* = 0.003).

## Discussion

Patients living with frailty (CFS score 5 to 8) had higher revisit rates within 30 days (17% vs. 11%) and 90 days (19% vs. 12%) compared to those living without frailty. They were also admitted to in-hospital care at a greater rate (57% vs. 47%) and had a significantly higher overall mortality rate (17.1% vs. 4.6%). Among patients living without frailty, those who made a revisit had a slightly higher mortality rate than those with only an index visit (5% vs. 2%).

Previous research done in the field of revisits (or unscheduled return visits) regarding older patients have mainly focused on comorbidity and the biological age, and has not incorporated frailty as a factor [26–30]. Tools traditionally used in the ED to assess patients physiological processes, like National Early Warning Score 2, 3-level-triage or Charlson Comorbidity Index perform poorly in predicting if older patients will return to the ED [31, 32]. It has been suggested that revisits or unscheduled return visits by older patients to the ED is a complex subject [33], and that frailty can be another factor to regard since it gives yet another dimension to revisits [34]. Previous research about revisits also focus mainly on the initial 72 h, 7 days or at most 30 days after the index visit [26, 28–30, 35]. By choosing the cut-off period to be 90 days, we made it possible to see patterns at a greater perspective and clarify possible patterns in revisiting patients, regarding if they were living with frailty or not. Since there still remains a gap in available research regarding revisits and frailty, determining the optimal follow-up period is difficult. Short-term follow-up can give information regarding if correct care was given and the right decisions were made at the index visit. However, to fully understand all revisits one would need to have a longer follow-up period.

Patients living with frailty seem to have more complex reasons for revisits, connected to social and/or environmental factors [36]. Regarding social community care services, previous research is not conclusive. Some say that having community care services increases the risk of revisits [37], while others have found that revisits increase if the patients do not have community care services [10, 36]. Since the current study does not take into consideration the patient’s use of community care services, this would be interesting to investigate further, as we saw in the current study that the group of non-frail patients was in the majority in both the index and revisiting groups. A fair assumption to make is that patients living with frailty tend to utilize some kind of community care service, while patients living without frailty do not. Results also indicate a connection between revisits per se and increased mortality, regardless if the patient is living with or without frailty. This coincides with a previous study from Sweden [10], which showed the number of visits to ED/healthcare increased during the last year of living. It would be of value to further research this phenomenon in regard to CFS-assessed patients, since it could lead to both optimizing patients end-of-life-care, as well as make sure that patients are being tended to in the right part of the chain of care.

Within the first 7 days following the index visit, revisit rates were comparable between patients living with or without frailty. Both groups demonstrated relatively low rates of revisits during this initial week. It has been shown previously that frailty cannot be linked to a higher rate of revisits within 72 h of the index visit [26, 35]. In the following revisit period (between day 8 to 90) from index visit, we saw a steady increase in the number of revisits, especially among patients living with frailty. A similar number of revisits of older patients (> 75 years of age, not necessarily frail) within 90 days has been reported previously [37]. Our results suggest that patients living with frailty get satisfactory care at the ED index visit, but that their healthcare needs may not be fully met outside of the hospital, necessitating revisits and often admission for in-hospital care [37].

Patients living with frailty demonstrated higher revisit rates compared to those without frailty, particularly between 8 and 30 days post-index visit, with an even more pronounced increase from day 31 to 90. This trend persisted despite the higher overall mortality rate in the group living with frailty. These findings suggest that assessing frailty using CFS could enhance both patient care and the emergency department’s ability to predict revisit frequencies.

An array of interventions to decrease revisits among older and/or patients living with frailty have been investigated previously in different ED settings. A geriatric management during in-hospital care, combined with an interdisciplinary transitional care intervention has been shown to reduce ED revisits and readmission to in-hospital care [36, 38]. Here, CFS could be useful as a tool to assess frailty, and to assure that the assessments of frailty become more uniform throughout the healthcare system. For the non-admitted patients, a comprehensive geriatric assessment and resulting adapted multifactorial interventions, could potentially reduce the number of revisits to the ED [39, 40]. This would require a more systematic collaboration between the ED, primary healthcare and community care services, than what is the case today in the current setting of this study. Primary healthcare would also have to develop strategies to increase availability to frail patients. Limited accessibility to primary care physicians is a known factor contributing to older patients making a revisit to the ED [37].

### Strengths and limitations

This prospective observational study was multicentered, which gave us an overall picture of revisits for older patients, assessed for frailty with CFS, from different sized hospitals in both rural and urban settings. This most likely makes the results of the study generalizableto a Swedish context. One possible limitation of the study was the lack of a clear distinction made between patients who got admitted at their index visit and those who were treated at the ED and discharged without in-hospital stay. The patients admitted at their index visit could possibly have greater health issues, and therefore be more prone to revisiting the ED. Another possible limitation of this study is the exclusion of approximately 50% of the eligible patients due to the absence of CFS-score at their index visit. Two related studies [22, 41] based on the same cohort as this study indeed provide some insight into this issue; One of our previous studies regarding the feasibility of CFS in the ED [22] identified high workload, critical illness, and staff oversight as the primary reasons for patients not receiving a frailty assessment. Additionally, there may be unintentional selection bias in the assessment process. Our second study [41], showed that eligible patients who were not assessed had a slightly lower mean age, similar to that of patients living without frailty. Hence, we suspect that healthcare providers may be less inclined to assess younger patients or those appearing robust, given that CFS is primarily designed to identify frailty rather than robustness.

We choose the term “revisit” in this study. However, there seems to be no clear consensus on the terminology within the field.

## Conclusions

Our results indicate that patients living with frailty make more revisits, are more often admitted at their revisit, and have an overall higher mortality rate than patients without frailtywithin 90 days of making an index visit to the ED. The occurrence of a revisit also seems to signal a risk for increased mortality, regardless if the patient is considered frail or not. Further studies should focus more in-depth on the patients’ reasons for making a revisit. Possible connections to the index visit, and whether the revisit could have been prevented, and how revisits affect patients provide other avenues for inquiry.

## Data Availability

The datasets used and/or analysed during the current study are available from the corresponding author on reasonable request.

## Abbreviations

ED: Emergency Department
CFS: Clinical Frailty Scale
